# Dexamethasone is Associated With a Lower Risk of the Progression of Thoracic Aortic Calcification in Breast Cancer Survivors

**DOI:** 10.3389/fphar.2021.740815

**Published:** 2021-12-10

**Authors:** Juan Lei, Aiting Liu, Yujia Ma, Guangzi Shi, Feng Han, Wenlong Jiang, Yongqiao Zhou, Chao Zhang, Yimin Liu, Xiaobo Huang, Hui Huang, Jie Chen

**Affiliations:** ^1^ Department of Cardiovascular, Sun Yat-sen Memorial Hospital, Sun Yat-sen University, Guangzhou, China; ^2^ Department of Cardiovascular, The Eighth Affiliated Hospital, Sun Yat-sen University, Shenzhen, China; ^3^ Department of Radiotherapy, Sun Yat-sen Memorial Hospital, Sun Yat-sen University, Guangzhou, China; ^4^ Department of Radiology, Sun Yat-sen Memorial Hospital, Sun Yat-sen University, Guangzhou, China; ^5^ Department of Ultrasound, Sun Yat-sen University Cancer Center, Sun Yat-sen University, Guangzhou, China; ^6^ Department of Emergency, The Second People’s Hospital of Huadu, Guangzhou, China

**Keywords:** dexamethasone, radiotherapy, aorta volume, thoracic aortic calcification, breast cancer

## Abstract

**Background and Purpose:** Breast cancer survivors have an increased cardiovascular risk, and vascular calcification is the pathological basis of cardiovascular disease. Some factors that affect the progression of thoracic aortic calcification (TAC) in survivors are unclear, and this study aims to explore the relationship between dexamethasone or radiotherapy and the progression of TAC in survivors.

**Materials and Methods:** This study included 189 female patients with breast cancer, and they were divided into the progression and non-progression TAC groups. Radiation or dexamethasone doses, and related laboratory parameters were collected.

**Results:** The cumulative dose of dexamethasone was higher [40 (10–180) mg versus 180 (80–270) mg, *p <* 0.001], and the cycle was longer [4 (1–6) cycles versus 6 (4–8) cycles, *p <* 0.001] in the non-progression TAC group. The cumulative dose (*r* = −0.303, *p <* 0.001) and cycle (*r* = −0.357, *p <* 0.001) of dexamethasone were negatively correlated with the level of increased TAC Agatston scores in survivors. Logistic regression analysis showed that dexamethasone was a protective factor for the progression of TAC (*p* = 0.029, odds ratio = 0.263, 95% confidence interval = 0.08–0.872). However, there wasn’t significant relationship between radiotherapy, radiation dose, follow-up time and the progression of TAC (all *p* > 0.05). In addition, aorta volume was positively correlated with the level of increased TAC Agatston scores in intensity modulated radiation therapy (*r* = 0.460, *p <* 0.001).

**Conclusion:** Dexamethasone is associated with a lower risk of the progression of TAC in breast cancer survivors, and there’s no correlation between radiotherapy and progression of TAC, but the aorta volume may be a predictor of the severity of progression of TAC.

## Introduction

Breast cancer is one of the most common cancers in the world (Siegel et al., 2020). With the advancement of medical technology, the survival rate of breast cancer patients has increased (Early Breast Cancer Trialists’ Collaborative, G., 2005). At the same time, multiple studies have proved that breast cancer survivors had an increased risk of cardiovascular diseases (CVD) (Rutqvist and Johansson, 1990; Cuzick et al., 1994; Chalker et al., 2004; Clarke et al., 2005; Hooning et al., 2007; Darby et al., 2013; Atkins et al., 2019). As an important pathological basis of CVD, vascular calcification (VC) increases the stiffness of the vascular wall and reduces the diameter, thereby increases the incidence of adverse cardiovascular events (Paloian and Giachelli, 2014; Kalsch et al., 2019). Computed tomography (CT) and Agatston scores are used to assess the occurrence and severity of VC, thus these help to clinicians identify patients with high risk of CVD (Ichii et al., 2013; Desai et al., 2018). However, some factors that affect thoracic aortic calcification (TAC) in survivors are unclear. Radiotherapy is a common treatment for breast cancer patients and has been observed to promote coronary and aorta calcification in patients with Hodgkin lymphoma (Apter et al., 2006). Glucocorticoid is frequently used as adjuvant drugs in chemotherapy or endocrine therapy in breast cancer patients, and studies have found that glucocorticoid promoted VC *in vivo* and *in vitro* (Kirton et al., 2006; Tinggaard et al., 2020). Furthermore, dexamethasone is the most commonly type of glucocorticoid in cancer therapy. So as to explore the relationship between radiotherapy or dexamethasone and the progression of TAC in breast cancer survivors, we conducted a cross-sectional study, then provided references for clinicians to prevent and delay the progression of TAC during the treatment.

## Materials and Methods

### Study Population

**FIGURE 1:**
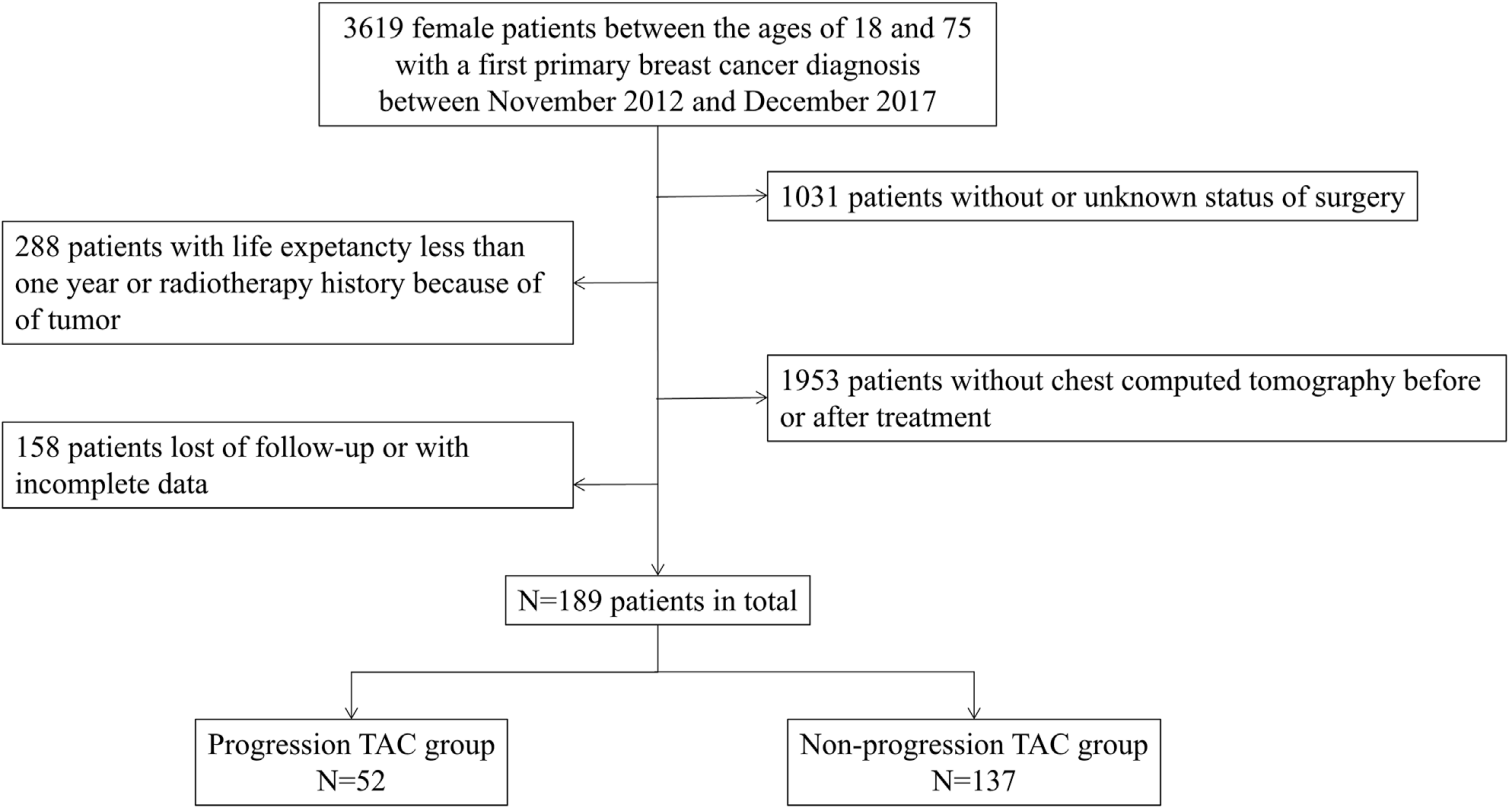
Flowchart of study. TAC indicates thoracic aortic calcification.

This was a single-center, retrospective, case-controlled study conducted in Sun Yat-sen Memorial Hospital of Sun Yat-sen University. Inclusion criteria were histologically confirmed breast cancer and imaging data according to the current guideline (Bevers et al., 2018). Therefore, a total of 3619 female patients, aged between 18 and 75, were included in this study, who were diagnosed with breast cancer for the first time between November 2012 and December 2017. Then following patients were excluded: 1) without or unknown status of surgery; 2) without chest CT pre- or post-treatment; 3) with life expectancy less than 1 year because of late stage of disease; 4) previous history of tumor or radiation therapy; 5) with history of long-term use of glucocorticoid; and 6) loss of follow-up or had incomplete data. Finally, 189 female patients were included in the cohort, and they were divided into the progression TAC group (the level of increased TAC Agatston scores>0) and the non-progression TAC group (the level of increased TAC Agatston scores = 0) according to the level of increased TAC Agatston scores by comparing pre- and post-treatment (Figure 1).

The study protocol conformed to the ethical guidelines of the 1975 Declaration of Helsinki by the Ethics Committee of Sun Yat-sen Memorial Hospital of Sun Yat-sen University (SYSEC-KY-KS-2020-101). The Ethics Committee waived the requirement for written informed consent because of its retrospective design.

### Data Collection

Data regarding medical history (hypertension and diabetes) and blood pressure, or biochemical parameters [phosphorus, uric acid, triglyceride (TG)], were abstracted from medical files. Tumor information (including location, stage, pathology), and treatment information (medicines for internal disease, chemotherapy, and dexamethasone), were collected. Radiation charts for patients who received radiotherapy were obtained. The information of death was abstracted from our follow-up system.

### Multidetector CT Analysis and Agatston Scores for TAC

Measurement of TAC was performed on a 64-row CT scanner. All the scans were read by the same 64-row CT scanner (Sensation 64, Siemens Medical Solutions, Erlangen, Germany) in the radiological department of our unit, and TAC above the diaphragm was used for analysis. Then, the CT calcification score statistical software V1.0 (China, Software copyright: 2020SR1269748) was used to calculate the total number of pixels within the regions around the identified calcifications to obtain the Agatston score. All the TAC data were analyzed by experienced radiologist who were blinded to the exact clinical information.

### Radiotherapy Mode and Dose

There were 65 patients treated with 2-dimensional radiation therapy (2D-RT). The chest wall received a dose of 45.0–50.4 Gray (Gy) in 25–28 fractions, and a boost dose about 10.0–16.0 Gy in 5–8 fractions to the tumor bed was recommended in patients at higher risk for recurrence. All 2D-RT treatments were delivered by Siemens Primus-H linear accelerator.

There were 72 patients treated with intensity modulated radiation therapy (IM-RT). The chest wall received a dose of 45.0–50.4 Gy in 25–28 fractions, and a boost dose about 10.0–16.0 Gy in 5–8 fractions to the tumor bed was recommended in patients at higher risk for recurrence. All IM-RT treatments were delivered by Varian Trilogy and Elekta Infinite linear accelerator. The maximum, minimum and mean radiation dose and aorta volume were analyzed by experienced radiotherapists who were blinded to the exact clinical information.

### Calculation of Cumulative Dose and Cycle of Dexamethasone

We excluded patients with a long-term history of taking glucocorticoid. We found that the type of glucocorticoid used by all patients was dexamethasone, and dexamethasone was used only by oral or intravenous injection in this study. The cumulative dose of dexamethasone was calculated during the entire follow-up process, and the cycle of dexamethasone was calculated by the cycle of dexamethasone used during chemotherapy, endocrine therapy or radiotherapy.

### Statistical Analysis

Variables are represented as means ± standard deviation and median (quartile spacing), or absolute numbers and percentage. Baseline variables were compared with independent *t* tests after performing Levene’s test or Mann Whitney test. *X* ^2^ or Fisher’s exact test was used for categorical variables, and Spearman correlation analysis was used to explore the relationship between influence factors and increased TAC Agatston scores. Logistic regression (conditional forward) was used to find out possible factors affecting the progression of TAC. All parameters showing a significant univariate relation with TAC were included as covariates, odds ratios (OR) and 95% confidence interval (CI) were calculated. Statistical Package for the Social Sciences (SPSS) software for Windows 25 (IBM Corporation, Armonk, New York, United States) was used for all statistical analyses. Significance was established at two-tailed *p* < 0.05.

## Results

### Comparison of Demographic and Baseline Clinical Characteristics Between the Progression TAC and Non-progression TAC Groups

During an average follow-up of 17.5 (6–80) months, 189 female breast cancer survivors were enrolled in total, including 52 (27.5%) survivors of progression TAC group and 137 (72.5%) survivors of non-progression TAC group. The progression TAC group was older, and the levels of phosphorus, blood urea nitrogen and TG were higher than those in the non-progression TAC group (all *p* < 0.05). However, there were no significant differences in diabetes, calcium, tumor location (left), stage and pathological calcification (all *p* > 0.05) (Table 1 and Supplementary Table S1). Interestingly, although there was no difference in the use of dexamethasone between the two groups (41 [78.8%] versus121 [88.3%], *p* = 0.096), the cumulative dose of dexamethasone was higher (40 [10–180] mg versus 180 [80–270] mg, *p <* 0.001) and the cycle was longer (4 [1–6] cycles versus 6 [4–8] cycles, *p <* 0.001) in the non-progression TAC group. In addition, there were no significant differences in the use of radiotherapy (33 [63.5%] versus 104 [75.9%], *p* = 0.087), radiation dose (34.0 ± 26.5 Gy versus 40.6 ± 23.6 Gy, *p* = 0.117) and follow-up time (16.8 ± 8.0 months versus 17.7 ± 13.0 months, *p* = 0.501) between the two groups (Table 1).

**TABLE 1:** Demographic and baseline clinical characteristics of patients.

Variables	Progression TAC Group	Non-progression TAC group	*p* value
	*n* = 52	*n* = 137	
Demographic characteristics			
Age, y	55.2 ± 8.0	45.3 ± 9.0	<0.001
BMI, Kg/m2	23.9 ± 3.2	23.0 ± 3.8	0.180
Hypertension, n (%)	13 (25.5)	8 (5.8)	<0.001
Diabetes, n (%)	4 (7.7)	6 (4.4)	0.364
Biochemical parameters			
FBS, mmol/L	5.7 ± 2.0	5.4 ± 1.2	0.129
Ca, mmol/L	2.3 ± 0.2	2.3 ± 0.1	0.600
P, mmol/L	1.3 ± 0.2	1.2 ± 0.2	0.032
Uric acid, mmol/L	355.2 ± 85.3	311.1 ± 73.8	0.002
CHOL, mmol/L	5.3 ± 1.1	4.9 ± 1.0	0.025
TG, mmol/L	1.7 ± 1.1	1.3 ± 0.7	0.007
LDL, mmol/L	3.6 ± 0.9	3.2 ± 0.7	0.005
THERAPY			
Statins, n (%)	1 (1.9)	0 (0)	0.104
Trastuzumab, n (%)	16 (30.8)	37 (27.0)	0.607
Anthracyclines, n (%)	30 (57.7)	90 (65.7)	0.308
Dexamethasone, n (%)	41 (78.8)	121(88.3)	0.096
Cumulative dose of dexamethasone, mg	40 (10–180)	180 (80–270)	<0.001
Cycle of dexamethasone, cycles	4 (1–6)	6 (4–8)	<0.001
Radiotherapy, n (%)	33 (63.5)	104 (75.9)	0.087
Radiation dose, Gy	34.0 ± 26.5	40.6 ± 23.6	0.117
Follow-up time, months	16.8 ± 8.0	17.7 ± 13.0	0.501

Values are expressed as mean ± SD, median (interquartile range) or number (%). BMI, body mass index; BUN, blood urea nitrogen; Ca, calcium; CHOL, cholesterol; FBS, fasting blood sugar; Gy, Gray; LDL, low density lipoprotein; P, phosphorus; SBP, systolic blood pressure; TAC, thoracic aortic calcification; TG, triglyceride.

### Association Between the Cumulative Dose or Cycle of Dexamethasone and the Increased TAC Agatston Scores

**FIGURE 2:**
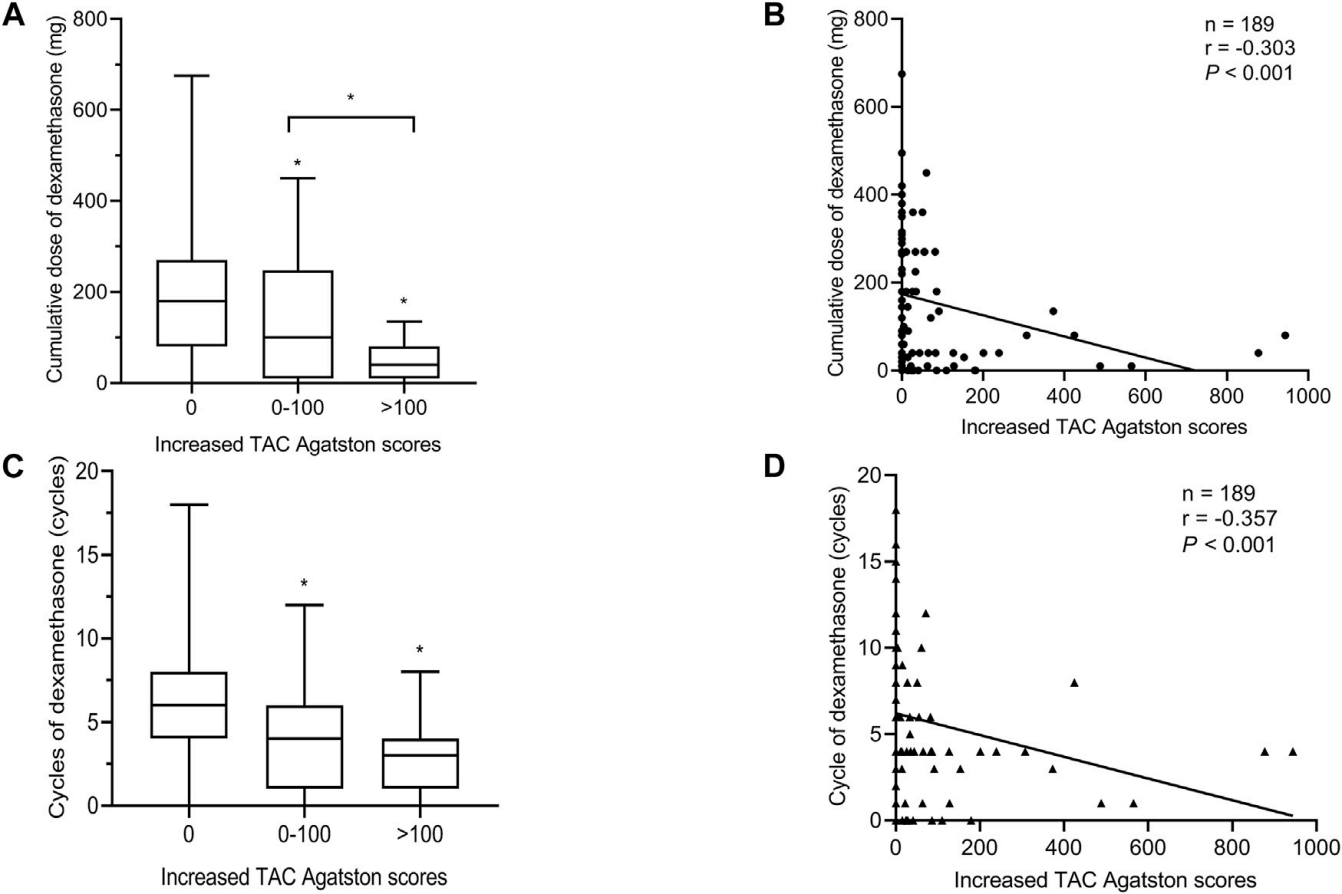
Relationship between the cumulative dose or cycle of dexamethasone and the increased TAC Agatston scores. Each symbol refers to one patient in Figures 2B,D. Boxplots showing median values (horizontal line inside the box), quartiles (box boundaries), and the largest and smallest observed values (lines drawn from the end of the box) in Figures 2A,C. **(A)** The cumulative dose of dexamethasone in different levels of increased TAC Agatston scores groups. *p < 0.05 versus the 0 or 0-100 group. **(B)** The cumulative dose of dexamethasone was negative correlation with the increased TAC Agatston scores (*r* = −0.303, *p <* 0.001). **(C)** The cycle of dexamethasone in different levles of increased TAC Agatston scores groups. *p < 0.05 versus the 0 group. **(D)** The cycle of dexamethasone was negative correlation with the increased TAC Agatston scores (*r* = −0.357, *p <* 0.001). TAC indicates. thoracic aortic calcification.

Comparing the cumulative dose and cycle of dexamethasone in different levels of increased TAC Agatston scores groups, as shown in Figures 2A,C, the group with a higher level of increased TAC Agatston scores was accompanied by a lower cumulative dose (median 180 versus 120 versus 40 mg, in 0, 0–100, and >100 groups, respectively, *p* < 0.05), and a shorter cycle (median 6 versus 4 versus 3 cycles, in 0, 0–100, and >100 groups, *p* < 0.05 versus the 0 group) of dexamethasone. Furthermore, there was a negative correlation between the cumulative dose (*r* = −0.303 *p <* 0.001, Figure 2B) or cycle of dexamethasone (*r* = −0.357, *p <* 0.001, Figure 2D) and increased TAC Agatston scores.

### Inconsistency of Radiation Exposure Dose and Locations of Progression of TAC

**FIGURE 3:**
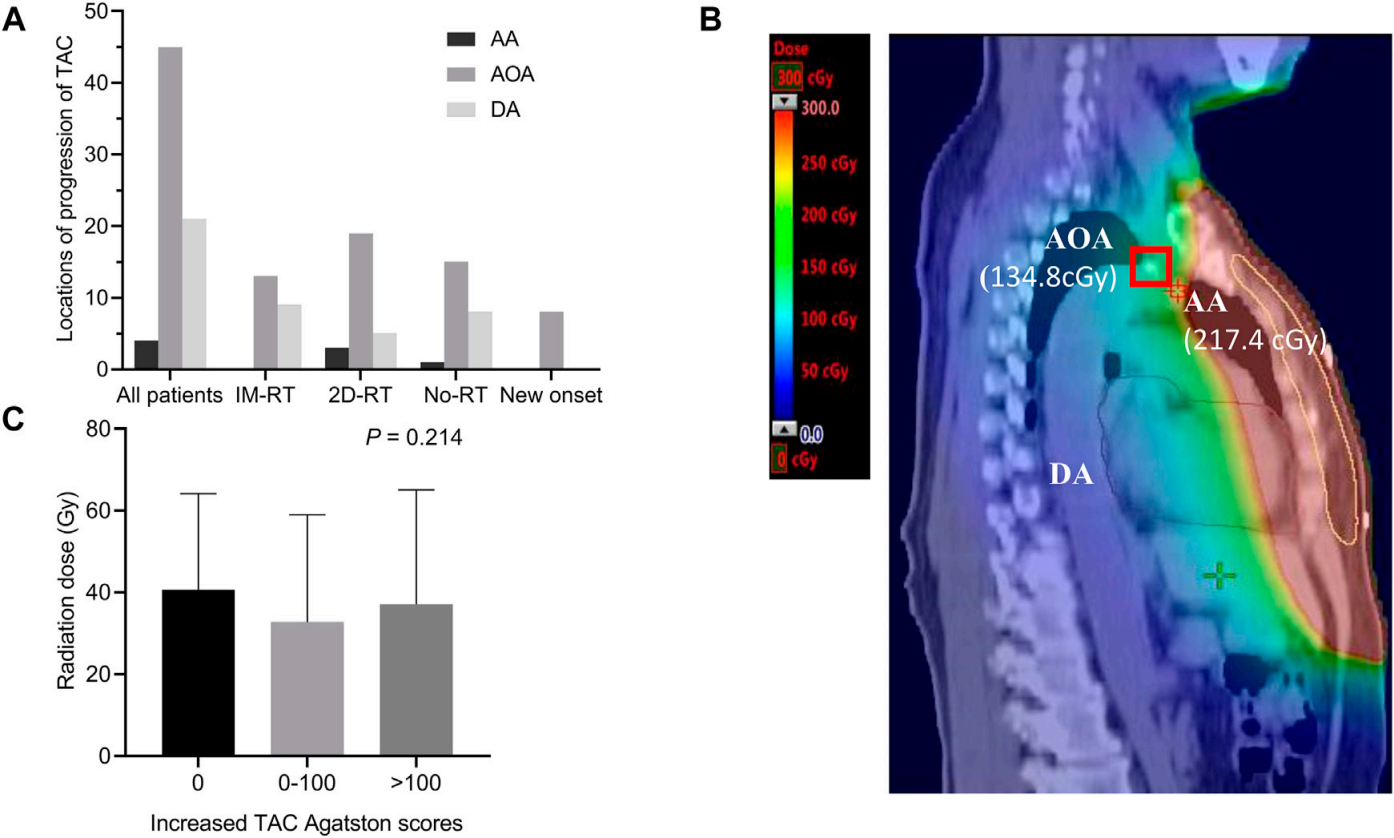
Inconsistency of radiotherapy exposure dose and locations of progression of TAC. **(A)** Locations of progression of TAC. The most common location of progression of TAC was the AOA, followed by DA and AA. Moreover, all new onset TAC were in the AOA. **(B)** CT of new onset AOA calcification in a female breast cancer survivor without TAC history. The radiation exposure dose of the thoracic aorta was displayed on CT, although the radiation exposure dose of AA was higher than that of AOA (217.4 versus 134.8 cGy), the new onset calcification occured at the AOA (marked with a red box) instead of AA. **(C)** Comparison radiation dose between different level of increased Agatston score groups. Histogram showing mean with standard deviation. AA indicates ascending aorta; AOA, arch of aorta; CT, computed tomography; DA, descending aorta; Gy, Gray; IM-RT, intensity modulated radiation therapy; No-RT, no radiation therapy; TAC, thoracic aortic calcification; 2D-RT, 2-dimensional radiation therapy.

The most common location of progression of TAC was the arch of aorta (AOA) in all breast cancer survivors, followed by descending aorta (DA) and ascending aorta (AA), and the same characteristics were seen in IM-RT, 2D-RT or no radiotherapy groups (Figure 3A). In addition, locations of all new onset TAC were the AOA in patients without TAC history. Figure 3B clearly showed that when breast cancer patients receiving radiotherapy, AA had the highest radiation exposure dose, followed by AOA and DA. However, the new onset TAC appeared in AOA instead of AA. In addition, there was no difference in the radiation dose between different levels of increased TAC Agatston scores groups (*p* = 0.214, Figure 3C).

### Characteristics of TAC Between IM-RT and 2D-RT Modes

No significant differences were observed in the level of TAC Agatston scores (all *p* > 0.05) and the number of TAC (13 [18.1%] versus 16 [24.6%], *p* = 0.348) between IM-RT and 2D-RT modes before radiotherapy. Although the follow-up time was longer in the 2D-RT group (15.5 ± 10.9 months versus 20.0 ± 13.3 months, *p* = 0.032), the levels of increased TAC Agatston scores and the number of TAC or progression of TAC after radiotherapy showed less differences (all *p* > 0.05). Moreover, there were no significant differences in the locations of progression of TAC after radiotherapy (all *p* > 0.05, Table 2).

**TABLE 2:** TAC characteristics of two radiotherapy modes.

Variables	IM-RT Group	2D-RT Group	*p* value
	*n* = 72	*n* = 65	
Follow-up time, months	15.5 ± 10.9	20.0 ± 13.3	0.032
Radiation dose, Gy	54.4 ± 6.2	52.6 ± 5.9	0.084
TAC before treatment			
TAC Agatston scores			
AA	0 (00)	0 (00)	0.066
AOA	0 (0–0)	0 (0–0)	0.284
DA	0 (0–0)	0 (0–0)	0.247
Total	0 (0–0)	0 (0–0.7)	0.393
TAC, n (%)	13 (18.1)	16 (24.6)	0.348
TAC after treatment			
TAC Agatston scores			
△AA	0 (0–0)	0 (0–0)	0.066
△AOA	0 (0–0)	0 (0–2.7)	0.070
△DA	0 (0–0)	0 (0–0)	0.743
△Total	0 (0–0)	0 (0–8.0)	0.170
TAC, n (%)	15 (20.8)	20 (30.8)	0.183
Progression of TAC, n (%)	13 (18.1)	20 (30.8)	0.082
AA, n (%)	0 (0)	3 (4.6)	0.208
AOA, n (%)	11 (15.5)	19 (29.2)	0.063
DA, n (%)	8 (11.3)	5 (7.7)	0.495
New onset, n (%)	2 (2.8)	4 (6.2)	0.585

Values are expressed as mean ± SD, median (interquartile range) or number (%). AA, indicates ascending aorta; AOA, arch of aorta; DA, descending aorta; Gy, Gray; IM-RT, intensity modulated radiation therapy; TAC, thoracic aortic calcification; 2D-RT, 2-dimensional radiation therapy.

### Influencing Factors for the Progression of TAC

**FIGURE 4:**
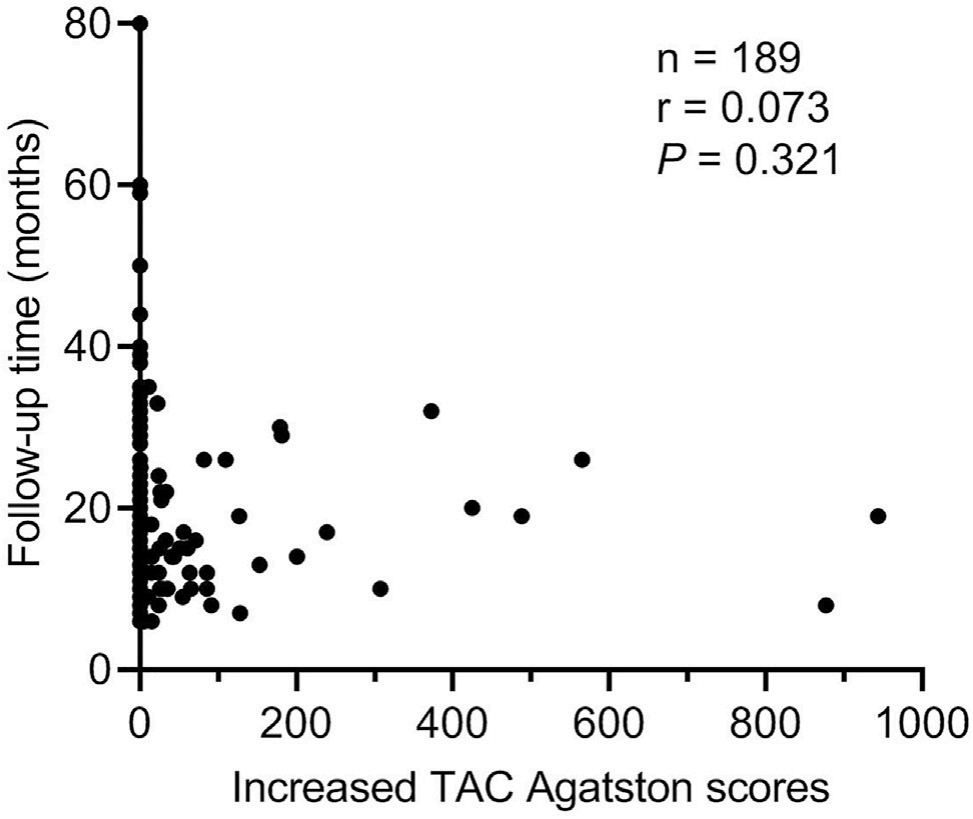
Correlation between follow-up time and increased TAC Agatston scores. Each symbol refers to one patient. TAC indicates thoracic aortic calcification.

The progression of TAC was used as the dependent variable, and some factors that might affect the progression of TAC were used as independent variables to perform logistic regression analysis (Table 3). All univariate factors that showed a significant relationship with the progression of TAC were included in the multivariate analysis (*p* < 0.15). Logistic regression analysis (conditionally forward) showed that dexamethasone was a protective factor for the progression of TAC (*p* = 0.029, OR = 0.263, 95% CI = 0.080–0.872), but there was no significant relationship between follow-up time and progression of TAC (*p* > 0.05). At the same time, Figure 4 also showed that there was no significant correlation between follow-up time and increased TAC Agatston scores (*p* = 0.321). Moreover, logistic regression analysis showed that baseline age (*p <* 0.001, OR = 1.149, 95% CI = 1.077–1.227), serum levels of phosphorus (*p* = 0.003, OR = 152.522, 95% CI = 5.481–4244.602) and TG (*p* = 0.007, OR = 2.006, 95% CI = 1.208–3.331) were independent risk factors for the progression of TAC in breast cancer survivors.

**TABLE 3:** Influencing factors for the progression of TAC.

Variables	Univariate analysis			*p* value	Multivariate analysis			*p* value
	OR	95% CI			OR	95% CI		
		Lower	Upper			Lower	Upper	
Demographic characteristics								
Age, years	1.140	1.088	1.194	<0.001	1.149	1.077	1.227	<0.001
BMI, Kg/m^2^	1.072	0.968	1.187	0.181	—	—	—	—
Hypertension	5.516	2.129	14.294	<0.001	—	—	—	—
Diabetes	1.819	0.492	6727	0.370	—	—	—	—
Biochemical parameters								
FBS, mmol/L	1.179	0.942	1.475	0.150	—	—	—	—
Ca, mmol/L	2.156	0.123	37.701	0.599	—	—	—	—
P, mmol/L	14.819	1.212	181.154	0.035	152.522	5.481	4244.602	0.003
Uric acid, mmol/L	1.007	1.002	1.012	0.004	—	—	—	—
CHOL, mmol/L	1.536	1.051	2.246	0.027	—	—	—	—
TG, mmol/L	1.731	1.122	2.670	0.013	2.006	1.208	3.331	0.007
LDL, mmol/L	1.972	1.210	3.214	0.006	—	—	—	—
THERAPY								
Dexamethasone	0.493	0.213	1.148	0.101	0.263	0.080	0.872	0.029
Tumor information								
Tumor location (left)	1.199	0.626	2.295	0.584	—	—	—	—
Tumor								
Pathological calcification	1.011	0.450	2.276	0.978	—	—	—	—
Follow-up time, months	0.993	0.965	1.021	0.612	—	—	—	—

Values are expressed as mean ± SD, or number (%). BMI, body mass index; Ca, calcium; CHOL, cholesterol; FBS, fasting blood sugar; Gy, Gray; LDL, low density lipoprotein; P, phosphorus; TAC, thoracic aortic calcification; TG, triglyceride.

### Aorta Volume was Positively Correlated with Increased TAC Agatston Scores in IM-RT Mode

**FIGURE 5:**
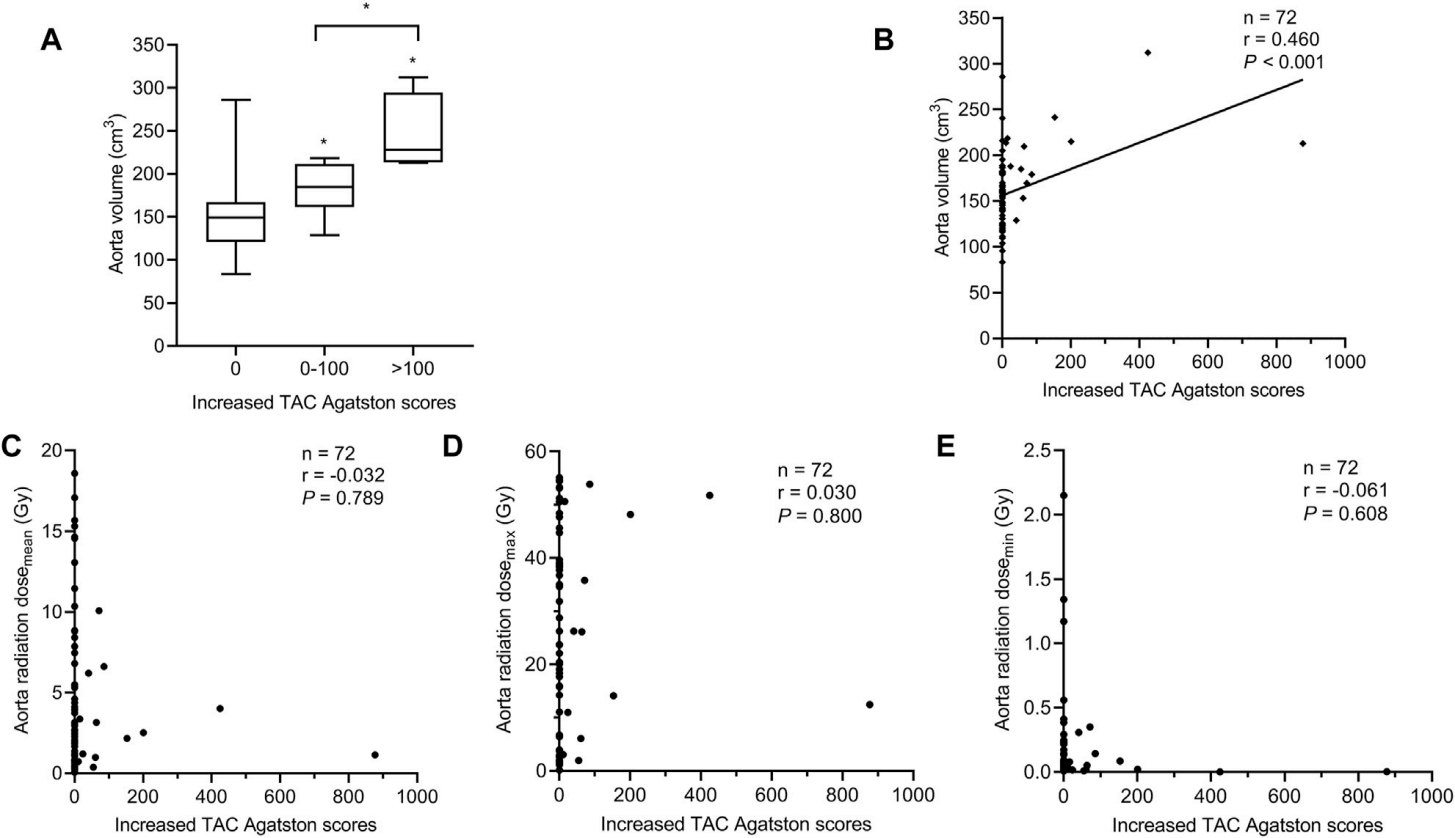
Relationship between the aorta volume and increased TAC Agatston scores in breast cancer patients with IM-RT. Each symbol refers to one patient in Figures 5B–E. **(A)** The aorta volume in different levels of increased TAC Agatston scores groups. Boxplots showing median values (horizontal line inside the box), quartiles (box boundaries), and the largest and smallest observed values (lines drawn from the end of the box). *p < 0.05 versus the 0 or 0-100 group. **(B)** The aorta volume was positively correlated with the increased TAC Agatston scores (*r* = 0.460, *p <* 0.001). **(C–E)**, Spearman correlation analysis showed that no significant correlation between aorta radiation dose_mean_ (*r* = −0.032, *p* = 0.789), dose_max_ (*r* = 0.030, *p* = 0.800), dose_min_ (*r* = −0.061, *p* = 0.608) and increased TAC Agatston scores. Dose_mean_ indicates mean dose; dose_max_, maximum dose; dose_min_, minimum dose; Gy, Gray; IM-RT, intensity modulated radiation therapy; TAC, thoracic aortic calcification.

Comparing aorta volume at different levels of increased TAC Agatston scores groups, data analysis showed that the group with a higher level of increased TAC Agatston scores was accompanied by a higher level of aorta volume (median 149.0 versus 184.8 versus 228.2 cm^3^, in 0, 0–100, and >100 groups, respectively, *p* < 0.05, Figure 5A). Moreover, Figure 5B showed a positive correlation between aorta volume and increased TAC Agatston scores (*r* = 0.460, *p <* 0.001). However, there was no significant correlation between the increased TAC Agatston scores and aorta radiation dose, regardless of the aorta radiation mean dose (*p* = 0.789), maximum dose (*p* = 0.800) or minimum dose (*p* = 0.608) (Figures 5C–E).

## Discussion

Major findings from our study revealed that the cumulative dose or cycle of dexamethasone was negatively correlated with the level of increased TAC Agatston scores, and dexamethasone was a protective factor for the progression of TAC in breast cancer survivors. On the contrary, there is no significant relationship between radiotherapy, radiation dose, radiotherapy modes or follow-up time and the progression of TAC. In addition, aorta volume was positively correlated with increased TAC Agatston scores, but no significant correlation was found with aorta radiation dose in IM-RT mode.

The risk of CVD is markedly increased in breast cancer survivors (Rutqvist and Johansson, 1990; Cuzick et al., 1994; Chalker et al., 2004; Clarke et al., 2005; Early Breast Cancer Trialists' Collaborative, G., 2005; Hooning et al., 2007; Darby et al., 2013). VC is the pathological basis of cardiovascular disease (Paloian and Giachelli, 2014; Kalsch et al., 2019), so finding out the possible factors that affect the progression of TAC in breast cancer survivors and conducting clinical intervention as soon as possible are more conducive to preventing and delaying the progression of TAC in breast cancer survivors.

Clinical data analysis showed that the cumulative dose and cycle of dexamethasone were fewer in progression TAC group. This was consistent with some clinical research results that lack of glucocorticoid promoted the development of calcification (Siebenmann, 1977; Klemm and Putzke, 1983; Halimi et al., 1986), and dexamethasone was found to inhibit the transformation of osteoblast phenotype *in vitro* (Chang et al., 1998; Iu et al., 2005; Park, 2012). This study also revealed that the level of increased TAC Agatston scores was negatively correlated with the cumulative dose and cycle of dexamethasone, and logistic regression analysis suggested that dexamethasone was a protective factor for the progression of TAC in breast cancer survivors. However, a study was found that intra-articular or intra-muscular injections glucocorticoid increased the level of coronary artery calcification scores (Tinggaard et al., 2020). In addition, Evans M et al. observed that higher cumulative prednisone dose was associated with the incidence of adverse cardiovascular events in rheumatoid arthritis patients (Evans et al., 2011; Karpouzas et al., 2020). In addition, it was observed in mouse that subcutaneous injection of prednisolone increased the deposition of calcium and phosphorus in the aorta (Helas et al., 2009). In this study, the way, dose and cycle of dexamethasone were different. During the treatment of breast cancer patients, dexamethasone was used for pretreatment before chemotherapy or adjuvant treatment of side effects of radiotherapy and chemotherapy, and it was mostly intravenous injected or oral. In the study of the relationship between rheumatoid arthritis and coronary artery calcification, rheumatoid arthritis patients were generally treated with intra-articular or intra-muscular glucocorticoid, and prednisone was injected subcutaneously in mouse. In addition, dexamethasone was mostly given in a short course of large doses intravenously or orally when undergoing chemotherapy or radiotherapy in this study, rather than long-term use in rheumatoid arthritis patients. Therefore, the cumulative dose of dexamethasone in breast cancer survivors was obviously lower than the cumulative dose of prednisone which was converted to dexamethasone in patients (Karpouzas et al., 2020). The cardiovascular toxicity caused by radiotherapy was not completely clear. At present, studies have confirmed that the cardiovascular toxicity caused by radiotherapy or chemotherapy was mainly due to the increased secretion of inflammatory factors, adhesion molecules and cytokines in heart and blood vessels, resulting in vascular endothelial injury and a series of subsequent pathological changes, and finally leading to the occurrence of diseases (Azimzadeh et al., 2011; Shukla et al., 2011; Mehta et al., 2018). As a widely known anti-inflammatory drug, dexamethasone may inhibit the progression of TAC through anti-inflammation in breast cancer survivors undergoing radiotherapy or chemotherapy (Brotman et al., 2005; Vandewalle et al., 2018). At the same time, the transformation of vascular smooth muscle cells to osteogenic phenotype was an important mechanism of vascular calcification, and dexamethasone may play a role by inhibiting the transformation (Chang et al., 1998; Iu et al., 2005; Park, 2012).

Coblentz C et al. first observed that radiotherapy promoted TAC in a patient who received radiotherapy for Hodgkin lymphoma in childhood (Coblentz et al., 1986), then other researchers observed that radiotherapy promoted coronary artery and aorta calcification in Hodgkin lymphoma patients with chest radiotherapy (Apter et al., 2006; Miki et al., 2020). However, this study found no correlation between radiotherapy and progression of TAC in breast cancer survivors which is consistent with previous studies (Chang et al., 2013; Soran et al., 2014; Takx et al., 2017). There are some reasons for the different results: Hodgkin lymphoma patients were treated with mediastinal radiotherapy, so the aorta was in the radiotherapy area and received more radiation exposure dose. Moreover, the follow-up time was longer than this study. In this study, breast cancer patients received chest wall radiotherapy, so the aorta wasn’t in the radiotherapy area, and the aorta received less radiation exposure dose with precise radiotherapy. Furthermore, the follow-up time was shorter, but it took decades to observe adverse radiotherapy events after radiotherapy (Clarke et al., 2005). In addition, comparing the TAC Agatston scores pre- and post-treatment could better reflect the relationship between radiotherapy and progression of TAC, but the above studies only observed the presence of vascular calcification. In the study of coronary calcium score in 12-years breast cancer survivors after adjuvant radiotherapy, researchers observed that no relationship between radiation dose and coronary artery calcification (Tjessem et al., 2015), so it supported the results of our study that there was no significant correlation between radiation dose and progression of TAC. Although logistic regression analysis found that there was no significant relationship between radiation dose and progression of TAC in all survivors, and no significant correlation between aorta radiation dose and increased TAC Agatston scores was observed in IM-RT mode. However, there was a positive correlation between the aorta volume and the increased TAC Agatston scores, and it’s consistent with the result that the coronary artery volume was a predictor of calcification risk in IM-RT mode (Milgrom et al., 2019). In addition, results showed that there was no significant relationship between follow-up time and progression of TAC. This is consistent with the findings of Kim B et al. who observed that radiotherapy did not change the incidence of common carotid artery calcification, although common carotid artery calcification was observed in one-third of patients receiving radiotherapy. No significant relationship was seen between the follow-up time and the occurrence of common carotid artery calcification (Markman et al., 2017; Kim et al., 2018). Furthermore, there was no significant difference in the characteristics of progression of TAC between IM-RT and 2D-RT modes, and the progression of TAC locations were AOA and DA. This is consistent with the study of Craiem D et al. (Craiem et al., 2014; Desai et al., 2018).

At the same time, results showed that baseline age, serum levels of phosphorus and TG were independent risk factors for the progression of TAC in breast cancer survivors, and multiple studies support our results (Kwak et al., 2014; Tesauro et al., 2017; Niu et al., 2019).

### Study Limitations

First of all, this study was a non-random, single-center retrospective study. We speculated on the observed association but couldn’t assess the causal relationship, and the mechanism couldn’t be explored. Second, the number of participants in our study was limited, and the average follow-up time was 17.5 months which was relatively short. Therefore, further randomized, prospective, long-term clinical studies with large samples are needed to verify our findings.

## Conclusion

We provide clinical evidence that dexamethasone is associated with a lower risk of the progression of TAC in breast cancer survivors, and there is no correlation between radiotherapy and the progression of TAC, but the aorta volume may be a predictor of the severity of progression of TAC. Moreover, baseline age, serum levels of phosphorus and TG are related to the progression of TAC. Our research provides references for clinicians to prevent or delay the progression of TAC by controlling related risk factors and appropriately using dexamethasone during the treatment and follow-up of breast cancer patients. However, it is still necessary to conduct further research to confirm that dexamethasone delays the progression of TAC.

## Data Availability

The raw data supporting the conclusions of this article will be made available by the authors, without undue reservation.
