# Supplementary material for: Dexamethasone is Associated With a Lower Risk of the Progression of Thoracic Aortic Calcification in Breast Cancer Survivors
Source: Front Pharmacol. 2021 Dec 10;12:740815. doi: 10.3389/fphar.2021.740815 (PMC8709127; doi:10.3389/fphar.2021.740815)
Supplement: Supplementary file 1 [file Table1.docx]

**Supplementary Table 1.** Baseline clinical characteristics of patients.

| Variables | Progression TAC Group | Non-progression TAC group | *P* Value |
| --- | --- | --- | --- |
|  | n = 52 | n = 137 |  |
| Biochemical parameters |  |  |  |
| ALP, U/L | 75.7 ± 25.0 | 68.1 ± 27.8 | 0.120 |
| BUN, mmol/L | 4.7 ± 1.2 | 4.3 ± 1.1 | 0.018 |
| Creatinine, μ mol/L | 71.9 ± 13.8 | 70.2 ± 11.4 | 0.423 |
| Tumor information |  |  |  |
| Location (left), n (%) | 30 (57.7) | 85 (62.0) | 0.584 |
| Stage |  |  | 0.349 |
| Stage Ⅰ, n (%) | 5 (9.6) | 24 (17.5) | - |
| Stage Ⅱ, n (%) | 25 (48.1) | 71 (51.8) | - |
| Stage Ⅲ, n (%) | 9 (17.3) | 21 (15.3) | - |
| Stage Ⅳ, n (%) | 13 (25.0) | 20 (14.6) | - |
| Pathological calcification, n (%) | 11(27.5) | 30(27.3) | 0.978 |
| Lymphatic metastasis, n (%) | 39 (75.0) | 92 (67.2) | 0.296 |
| Organ metastasis,  n (%) | 13 (25.0) | 20 (14.6) | 0.093 |
| ER positive, n (%) | 40 (76.9) | 101 (73.7) | 0.507 |
| PR positive, n (%) | 24 (46.2) | 75 (54.7) | 0.291 |
| Her-2 positive, n (%) | 10 (22.2) | 25 (22.3) | 0.989 |

Values are expressed as mean ± SD, median (interquartile range) or number (%). ER, estrogen receptor; Her-2, human epidermal growth factor receptor-2; PR, progesterone receptor.
